# The Turning-Point in the Hospital Campaign

**Published:** 1920-04-10

**Authors:** 


The Hospital

The Workers' Newspaper of Administrative Medicine and Institutional Life, Administration, National Insurance and Health.

No. 1766, Vol. LXVIII. SATURDAY, APRIL 10, 1920. PRICE TWOPENCE

### THE TURNING-POINT IN THE HOSPITAL CAMPAIGN.

The financial campaign on behalf of the voluntary hospitals is now in the critical condition of a movement which has been carried near success at many isolated points, at the extremes, as it were, of the line of attack, but which has not been pushed home at the centre. Consequently, an effort not made now may endanger all that has been gained. The strong point is in the provinces, the weak spot is in London. This fact should not be missed. It is little use for the branches to put forth leaves if the sap is failing in the old trunk; and that the sap is thought to be failing there is believed by Lord Knutsford, who has now committed himself to a policy of State aid. He proposes that the State should undertake to provide one-third of the cost of maintenance of approved hospitals. Leaving aside for a moment a criticism of this policy, we merely use it as an illustration that the campaign is as weak at the centre as it is prospering at the wings. We cannot, alas! build every hope upon the latter, because history teaches that the Roman Empire perished at the centre and that the provinces, which were much more ignorant than the capital, soon collapsed after the centre had deteriorated. The same lesson may apply equally to the voluntary system. The right policy, then, must be to prosecute the financial campaign in the provinces with even greater vigour, and to do all that can be done in London to repair the relative failure there. The provincial hospitals are valuable not only for their success, but for their example; and if their success can be made complete it may react favourably on opinion in London, and therefore be of incalculable benefit to the whole voluntary system. We do urge the provincial hospitals, therefore, to follow the success already achieved at Cardiff, Leeds, and Nottingham, and to pursue the abundant good beginnings which we have recorded week by week all over the country. The vitality is great, and might be made abounding. The opportunity is within their grasp. The men only are needed to make the most of it.

The reason why the efforts of the provinces have been more successful than those in London is, as Lord Knutsford well put it the other day, that there is more *esprit de corps* in the provinces. We admit the fact, but suggest that Lord Knutsford hardly drew the right inference from it. *Esprit de corps* is essential to any voluntary system, but the *esprit* cannot exist without the *corps*. Consequently, the problem is to give body to the existing *esprit* in the provinces, and to create the *esprit* by providing form and body to the amorphous multitudes of London. It is the latter point which London chairmen seem to miss. They appeal to all and sundry, because they have no body of supporters behind them. They even declare, in effect, that no such body can be formed. This we do not believe. On the contrary, we suggest that to create such a body is the right policy, and that the main effort should now be directed to that end, and no longer dissipated in all directions. After all, the London hospitals have been chiefly maintained by the bodies of subscribers which have been created out from the amorphous crowd. King Edward's Hospital Fund for London is a centre of attraction, mainly for bequests. It represents in principle a body of testators, to which intending testators often give during their lives. The Hospital Sunday Fund represents the body of church-goers. It is a denominational corps. The Hospital Saturday Fund is composed of the weekly wage-earners, though, of course, only a fraction of such folk, though a loyal and generous fraction, contributes to it. The League of Mercy, on the other hand, is a body of small subscribers, and has, perhaps, as much *esprit de corps* as any of these, if only because the element of personal service has been greater or more obvious in it. Now, if these facts mean anything at all, they mean two things—first, that the London hospitals are mainly supported by organised sections of the people, and, secondly, that the *esprit de corps* is strongest in London, where there exists a body of people united in a common aim. The lesson, therefore, is to leaven the mass still further by the creation of other bodies of similar type. What has been done in this direction can be done again, and the organisation extended to new classes. We suggest, therefore, that an alternative to State aid exists, and that those who believe in the voluntary system should set to work to try it. We cannot see why the banks, the city offices, the West End shops should not be approached for weekly collections. An effort should be made to organise these in every place where the Saturday Fund has no supporters. The field is wide, the method familiar. Side by side with this plan, the employers of the firms in which such collections are organised should be asked to contribute either in proportion to the number of their staffs or in proportion to the amount collected. If such a scheme is not discussed by the proposed conference of London hospitals, that conference will have neglected the nearest opportunity to hand. We suggest that the scheme would be better if it were made a joint request, because London is difficult to organise in the relatively small sections served by each hospital. We do not believe that the London offices, banks, and shops would refuse such a joint proposal, and would like to hear the opinion of such people as Sir Woodman Burbidge, of Harrods' Stores, and Mr. Gordon Selfridge upon it.

Meantime, let the provincial hospitals go ahead for all they are worth, not only for their own pride of achievement, but to save London from itself and the voluntary system generally. They have a great trust at this moment, and if they are true to it may have the reward of setting such an example as will lead the Government to say: "The State cannot differentiate. The country have shown that they need not burden the Treasury. The London hospitals are self-condemned if they do not follow suit."

